# LSU family members and NBR1 are novel factors that contribute to homeostasis of catalases and peroxisomes in *Arabidopsis thaliana*

**DOI:** 10.1038/s41598-024-76862-4

**Published:** 2024-10-25

**Authors:** Anna Niemiro, Konrad Jurczewski, Marzena Sieńko, Anna Wawrzyńska, Marcin Olszak, Jarosław Poznański, Agnieszka Sirko

**Affiliations:** Institute of Biochemistry and Biophysics Polish Academy of Sciences, Pawinskiego 5A St., 02-106 Warsaw, Poland

**Keywords:** Catalase, Peroxisome, LSU1, NBR1, N-BODIPY, Protein structure modelling, Molecular biology, Plant sciences

## Abstract

The short coiled-coil LSU (RESPONSE TO LOW SULFUR) proteins are linked to sulfur metabolism and have numerous protein partners. However, most of these partners lack direct links to sulfur metabolism, and the role of such interactions remains elusive. Here, we confirmed LSU binding to *Arabidopsis* catalase (CAT) and revealed that NBR1, a selective autophagy receptor, strongly interacts with LSU1 but not with CAT. Consequently, we observed the involvement of autophagy but not NBR1 in CAT removal. The *lsu* and *nbr1* mutants differed from the wild-type plants in size and the number of yellow fluorescent protein (YFP)-CAT condensates, the number of peroxisomes, and photosynthetic pigments levels in the presence and absence of stress. We conclude that LSU family members and NBR1 contribute directly or indirectly to CAT and peroxisome homeostasis, and the overall fitness of plants. Our structural models of CAT–LSU complexes show at least two regions of interaction in CAT, one of which is at the N-terminus. Indeed, the N-terminally truncated variants of CAT2 and CAT3 interact more weakly with LSU1 than their full-length variants, but the extent of reduction is higher for CAT2, suggesting differences in recognition of CAT2 and CAT3 by LSU1.

## Introduction

The *Arabidopsis thaliana* genome contains a family of four *RESPONSE TO LOW SULFUR* (*LSU*) genes, *LSU1* (At3g49580), *LSU2* (At5g24660), *LSU3* (At3g49570), and *LSU4* (At5g24655), which encode small proteins with a coiled-coil region^1^. The *LSU* genes have been associated with sulfur metabolism because they are strongly induced by a sulfur deficit^2^. They are also part of an OAS cluster (or an extended OAS cluster), namely the set of genes regulated by *O*-acetyl-L-serine^3,4^. Consistently, gene expression analyses of the *LSU* gene family in plants led to the conclusion that these genes have been evolutionarily conserved in angiosperms and that members of this family play an important role in the regulation of sulfur transport and assimilation^5^. Nevertheless, as demonstrated recently, they are not essential for plants: The quadruple *lsu1–4* deletion mutant (q-lsu-KO) is viable and not distinguishable from the wild-type (WT) when grown in soil in a growth chamber. Only at the stage of seedlings does the q-lsu-KO mutant have slightly shorter roots than the WT in sulfur-deficient medium^6^. Interestingly, *LSU* overexpression confers cadmium tolerance by modulating sulfur metabolism in at least two distinct places of the pathway: (1) inhibition of biosynthesis and promotion of aliphatic glucosinolate degradation, and (2) promotion of sulfur assimilation^7,8^.

The LSU proteins are involved in multiple protein–protein interactions, and the vast majority of their partners identified by high-throughput methods have no links to sulfur metabolism^9^. Two members of the family from *Arabidopsis*, LSU1 and LSU3, are part of an integrated network of host proteins targeted by plant pathogen effectors^10^. Subsequently, LSUs have been defined as functionally unclear stress-related hubs, and the interactome of LSU1, LSU3, and LSU2 protein partners have been constructed^11^. Some data suggest crosstalk between LSUs and phytohormones in tobacco^12^ and *Arabidopsis*^13^. Identification of the biological function of LSUs is complicated by the fact that their partners are localized in different cellular compartments and cannot be categorized into any specific biological process or molecular function. Only a few interactors have been validated by other methods, and there have been fewer attempts to assign biological functions to these interactions. There are two examples of chloroplast proteins where the biological consequences of their interactions with LSU family members have been characterized. The first example is RAP (At2g31890), an octotricopeptide RNA-binding protein, postulated to interact physically with LSU2 to negatively regulate host defense responses, which are positively regulated by LSU2^14^. The second example is a protein located in the chloroplast: The enzymatic activity of the iron-dependent superoxide dismutase FSD2 is stabilized by physical interaction with LSU1^15^. Notably, the positive involvement of LSU in reactive oxygen species (ROS) removal supports the above-mentioned postulated role of LSU proteins in mitigating environmental stress.

There are also some examples of verified LSU interactions with as-yet unidentified biological significance for any of the partners, including proteins such as catalase 2 (CAT2) from Arabidopsis^9^, a tobacco selective autophagy cargo receptor Joka2^16^, and its homologue from Arabidopsis NBR1 (NEXT TO BRCA1 GENE 1)^9^.

Catalases are the most abundant peroxisomal antioxidant enzymes involved in hydrogen peroxide (H_2_O_2_) removal. These enzymes catalyze a reaction that results in production of two H_2_O molecules and one O_2_ molecule from two H_2_O_2_ molecules^16^. Catalases are tetrameric enzymes^17^. *Arabidopsis* has three genes that encode catalase isoforms: *CAT2* is expressed in photosynthetic tissues, *CAT3* is associated with vascular tissues, and *CAT1* is expressed in seeds and reproductive tissues. Moreover, *CAT2* and *CAT3* are regulated by the circadian clock^18^. The CAT isoforms in Arabidopsis have a highly conserved amino acid sequence and the results of modeling of their 3D structures revealed a very similar arrangement of α-helices and β-strands^19^. All three isoforms have the C-terminally located non-canonical peroxisome targeting signal (PTS), and their import into peroxisomes depends on PEX5, a peroxisome import receptor that functions as a redox switch and retains catalase in the cytosol during oxidative stress^20^. Plant catalases have been detected not only in peroxisomes, but also in nucleus^21^ and cytosol. The nuclear interactors of catalases include the nucleoporin complex proteins NUP43 and NUP98 (suggesting that catalases are involved in nucleocytoplasmic transport) and proteins involved in epigenetic regulation. In the cytosol, plant catalases interact with numerous stress signaling proteins and can affect their involvement in different redox signaling pathways^22,23^. Interestingly, CAT3 functions as a transnitrosylase that specifically modifies *S*-nitrosoglutathione reductase (GSNOR), an enzyme responsible for the release of nitric oxide (NO) from *S*-nitroglutathione (GSNO, a major reservoir of NO) to regulate NO-based redox signaling in plants. *S*-nitrosylation of GSNOR1 at Cys-10 mediated by CAT3 (also known as repressor of GSNOR1 [ROG1]) induces local conformational changes and helps to target GSNOR1 for autophagic degradation. Thus, CAT3 and GSNOR1 may form a positive feedback loop to modulate the intracellular NO level^24,25^. The Cys-343 residue present in CAT3 but absent in CAT1 and CAT2 (both contain Thr at this position) is critical for the transnitrosylase activity^24^.

Peroxisomes play an important role in fatty acid β-oxidation and photorespiration and contribute to the synthesis of some phytohormones, ROS detoxification, and signaling. Peroxisomes are the main sites of H_2_O_2_ generation in plant cells. For this reason, peroxisomal catalase is the main H_2_O_2_ eliminator. Peroxisome homeostasis is maintained by coordination between their biogenesis and turnover. Peroxisomal proteins are synthesized in the cytosol and imported into peroxisomes in a complex process involving PEX5 and PEX7 as receptors of peroxisome-targeting signals (PTS1 and PTS2, respectively). PEX5 cycles between the peroxisome matrix and cytosol in a dynamically controlled way that requires its monoubiquitination and deubiquitination^26,27^. Pexophagy, a selective autophagy pathway to remove peroxisomes, plays a crucial role in peroxisome remodeling (during functional glyoxisome-peroxisome transitions of peroxisomal content) and in peroxisomal quality control^27^. NBR1, a selective autophagy receptor, has been identified as a specific receptor of pexophagy in mammals^28^. Although the involvement of NBR1 in pexophagy in plants has not been completely excluded, this protein is not necessary for pexophagy in *Arabidopsis*. These species can use an NBR1-independent pathway to target peroxisomes for autophagic degradation^29^. It is also unclear how plant peroxisomes are marked for degradation and which peroxisomal compound or protein could serve as a pexophagy marker.

In this study, we explored the biological significance of the interaction between LSU1 and NBR1 with catalases and peroxisomes. Then, we examined the amount, activity, and intracellular localization of catalases in WT plants and the mutants deprived of all four LSUs or NBR1. Our results confirm our previous observation in *Arabidopsis* that catalases and LSUs interact^9^. We also demonstrated that LSUs and the autophagy cargo receptor, NBR1, modulate the pattern of condensates of ectopically expressed yellow-fluorescent protein (YFP)-CAT2 and YFP-CAT3. Moreover, LSUs and NBR1 are presumably involved in maintaining the homeostasis of catalases and peroxisomes, particularly in stress conditions. In addition, we constructed a three-dimensional (3D) model of the CAT–LSU1 interaction that revealed LSU1 can bind to the N-terminus of catalases and binds to monomers rather than tetramers of catalases.

## Results

### Neither *LSU1* nor *NBR1* contribute to catalase activity and degradation

**Fig. 1:**
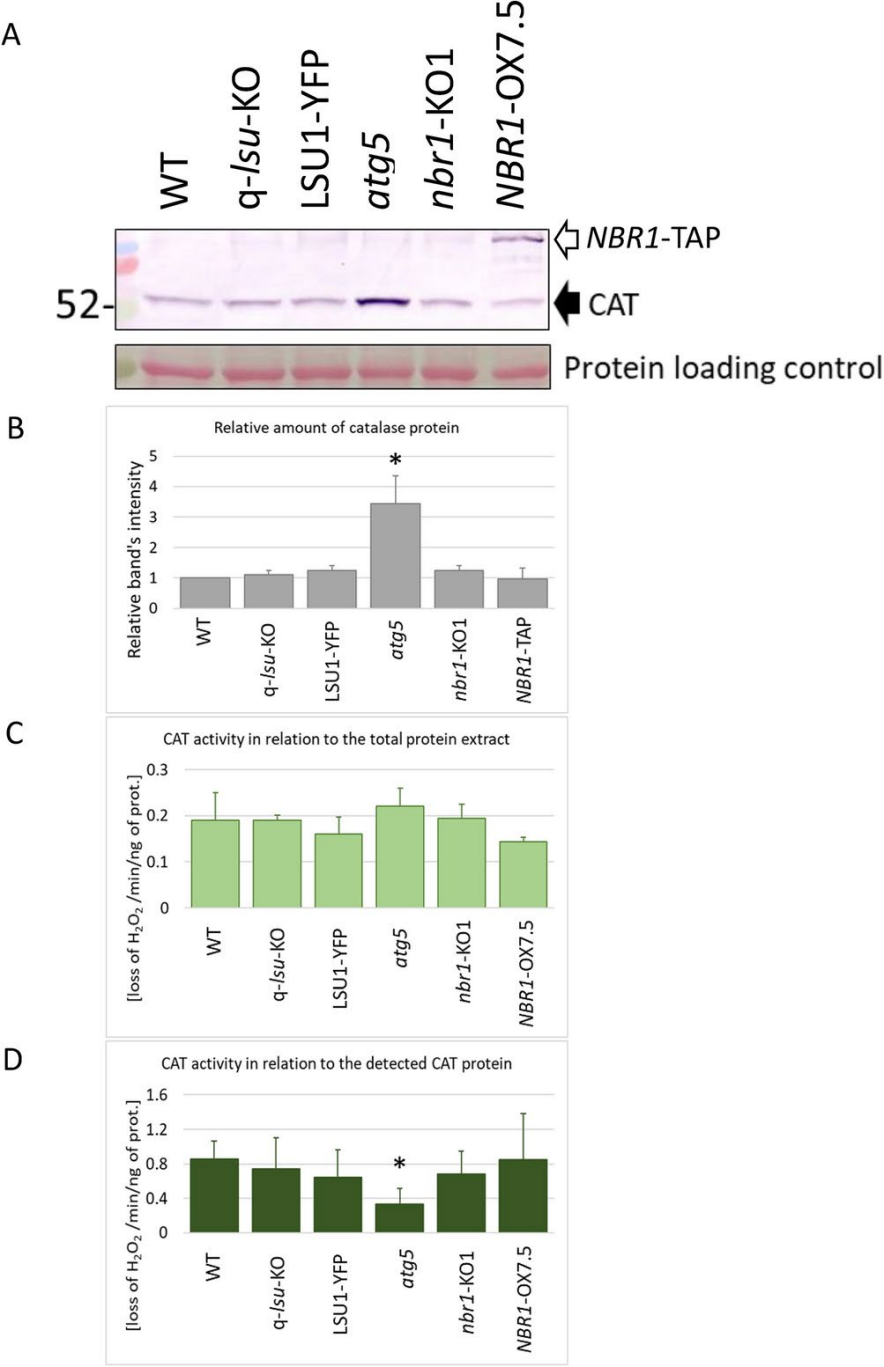
Monitoring the amount and activity of catalase. (**A**) Western blotting of total plant extracts. In *NBR1*-OX, the anti-catalase antibody detected two bands: the expected catalase band at 52 kDa (pointed by filled arrow) and a chimeric NBR1–TAP fusion proteins (the TAP-tag includes a fragment of an immunoglobulin heavy chain, causing some non-specific binding by the anti-catalase antibody; unfilled arrow). Ponceau S staining (protein loading control) confirmed equal protein loading across lanes based on Rubisco small subunit abundance. (**B**) Quantification of the catalase protein level. The relative amount of catalase was determined from western blots of three independent experiments. Catalase activity (H_2_O_2_ loss per min) was measured (**C**) per unit (ng) of total protein and (**D**) per unit (ng) of catalase protein in the extracts. The quantitative data come from three independent experiments, each in triplicate. The statistically significant differences from wilde type (WT) are marked with asterisks.

To examine the biological significance of the previously reported^9^ interaction between LSU family members and CAT2, we examined the amount and activity of catalase in the WT, q-*lsu*-KO, and *LSU1*-overexpressing plants. Additionally, to examine the involvement of autophagy in catalase degradation, we used the autophagy-deficient *atg5* mutant and the lines without the selective autophagy cargo receptor, NBR1 (*nbr1*-KO), and overexpressing NBR1 (*NBR1*-OX) as controls. There were no significant differences between the lines, except *atg5* had a higher amount of the catalase protein (Fig. 1). These results indicate that neither LSU nor NBR1 are essential for the control of catalase degradation or activity in the optimal, non-stressed conditions. Assuming that a threefold higher amount of catalase protein in the *atg5* line than in WT results from accumulation of defective catalase in this line, we calculated catalase activity in two ways, either based on the amount of total protein in the extract or based the amount of the catalase protein estimated in the same extracts via immune detection using an anti-catalase antibody. The catalase activity calculated based on the amount of catalase detected in the extract was about 2.5-fold lower in the *atg5* mutant than in the other lines (Fig. 1 and Fig. S1). Although we failed to demonstrate that either LSU1 or NBR1 are involved in controlling the amount or activity of catalase, we can speculate that autophagy is needed to remove defective catalase because the catalase protein is accumulated in the autophagy deficient *atg5* mutant. The second test, yellow fluorescent protein (YFP) cleavage, showed an increase (83% vs. 74%) in YFP release in the WT line after treatment with the TOR (target of rapamycin kinase) inhibitor, but the difference was not significant. Surprisingly, degradation of the YFP-CAT2 protein was significantly higher in the nbr1-KO mutant regardless of autophagy induction (Fig. S2). An explanation for this observation requires additional studies.

### The *lsu* and *nbr1* mutants have smaller YFP-CAT2 and YFP-CAT3 condensates than WT

**Fig. 2:**
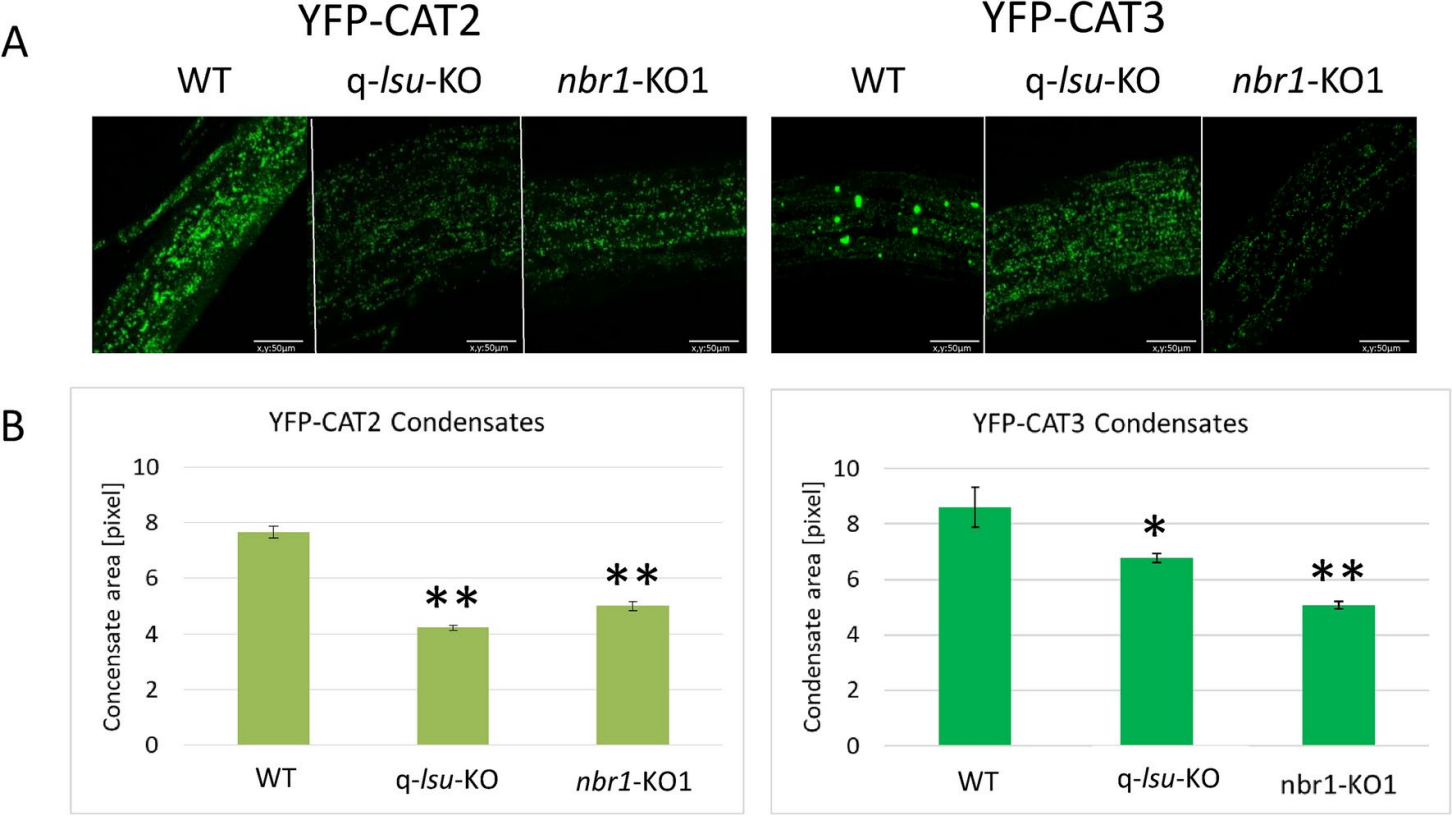
Comparison of the microscopic fluorescent condensates visualized in the roots of WT, q-*lsu*-KO, and *nbr1*-KO1 seedlings stably producing YFP-CAT2 and YFP-CAT3. (**A**) Representative micrographs (frames) from the analyzed plants. A frame is defined as a randomly selected the microscopic field with an area of 150 × 150 pixels within the root. Three randomly selected frames from the root area were used for the statistical analysis shown in B. Scale bars, 50 μm. (**B**) The mean area of condensates in the analyzed lines with the standard error indicated. The charts were plotted based on analyses of two independently obtained lines except YFP-CAT3 in q-*lsu*-KO, where only one line was available. Quantitative data were collected from biological replicates, with three frames per replicate. For YFP-CAT2 in WT, q-*lsu*-KO, and *nbr1*-KO1, the data are from 12, 9, and 6 frames, respectively. For YFP-CAT3, the data are from 18, 9, and 12 frames, respectively. The asterisks indicate a significant difference compared to the WT calculated by t-test (**p < 0.0001; *p = 0.015).

We decided to examine the role of LSU1 and NBR1 in the cellular localization of two major CAT isoforms, CAT2 and CAT3. We observed the area and number of YFP-CAT2 and YFP-CAT3 condensates in three genetic backgrounds, WT, q-*lsu*-KO, and *nbr1-*KO1 with fluorescent microscopy. The transgene encoding chimeric YFP-CAT2 and YFP-CAT3 proteins was controlled by the constitutive promoter and it is necessary to keep in mind that it is a non-native expression. Representative micrographs and the comparison of the mean area and the mean number of the condensates are shown in Fig. 2. For the WT background, the mean area of YFP-CAT2 and YFP-CAT3 condensates was larger than in the mutants. The lack of LSUs had a stronger effect on reducing the area of YFP-CAT2 than of YFP-CAT3 condensates compared with WT (to 55% and 79%, respectively). In contrast, the lack of NBR1 had a weaker influence on reducing the area of YFP-CAT2 than of YFP-CAT3 condensates compared with WT (to 65% and 59%, respectively). For the *nbr1*-KO1 line, the mean area of the YFP-CAT2 and YFP-CAT3 spots was similar, while the WT and q-*lsu*-KO lines showed slightly larger YFP-CAT3 spots than YFP-CAT2 (1.12-fold and 1.62-fold, respectively) (Fig. 2B).

### The *lsu*, *nbr1*, and *atg5* mutants have a tendency for accumulation of peroxisome aggregates in osmotically stressed seedlings

**Fig. 3:**
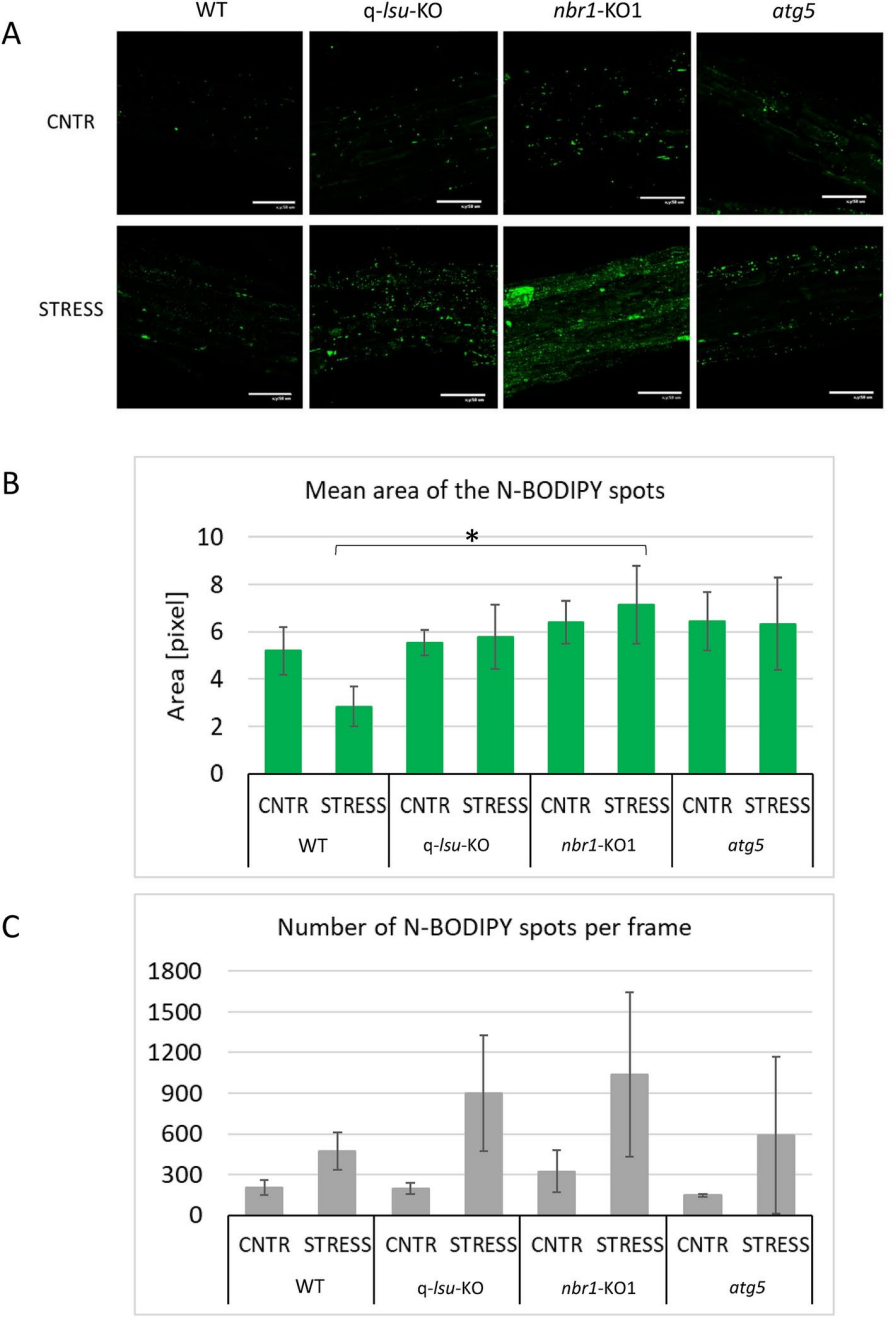
Visualization of peroxisomes by N-BODIPY in root tissues of the WT, q-*lsu*-KO, *nbr1*-KO1, and *atg5* seedlings in the absence and presence of osmotic stress. (**A**) Representative micrographs (frames) from the analyzed plants. Scale bars, 50 μm. (**B**) The mean area of the N-BODIPY spot with the standard error indicated. No statistically significant differences between the lines were found in the control conditions, while in the stress only the *nbr1*-KO mutant had significantly larger peroxisomes spots than the WT (marked by an asterisk) (**C**) The mean number of N-BODIPY spots per frame with the standard deviation indicated. For osmotic stress, seedlings were incubated for 3 h in 0.3 M NaCl; H_2_O denotes the absence of stress. The differences were not statistically significant due to high variation between the repetitions.

N-BODIPY (nitro-4,4-difluoro-4-bora-3a,4a-diaza-s-indacene) serves as an indicator of the level of peroxisomes in plants due to its ability to interact specifically with peroxisomes^30,31^. This compound has been used to demonstrate that osmotic stress elevates the amount of peroxisomes in plants^31^. We used N-BODIPY staining to compare the amount of peroxisomes in the WT, q-*lsu*-KO, *nbr1*-KO1 and *atg5* lines in the presence and absence of osmotic stress (Fig. 3). We included the *atg5* mutant as a control of the response to stress because peroxisomes damaged by oxidative stress are degraded selectively by autophagy, and aggregated peroxisomes accumulate in this autophagy-deficient mutant^32^. The *nbr1*-KO1 and q-*lsu*-KO mutants behave similarly to *atg5* relative to WT. This result is expected for NBR1 but novel for LSU and indicates the involvement of these proteins in the process of peroxisome homeostasis.

Interestingly, the mean area of the spots was reduced (1.8-fold) by the osmotic stress in the WT line, with a tendency to be larger in the other lines (Fig. 3B). The number of spots increased after stress in all lines; however, there were large differences between the biological replicates, as indicated by the large standard deviations, especially in the mutants and these changes were not statistically different (Fig. 3C). Although a lot of effort was put into getting the better reproducibility a huge variation was observed even in technical repetitions. Nevertheless, based on the tendency for larger and more numerous N-BODIPY-stained spots in the mutants than in the WT we speculate that the *atg5*, q-*lsu*-KO, and *nbr1*-KO1 mutants might accumulate peroxisome aggregates after the stress. Excessive aggregation may be related to impaired clearance due to the lack of NBR1 or LSU or defective autophagy.

### NBR1 localizes in the proximity of peroxisomes after oxidative stress

**Fig. 4:**
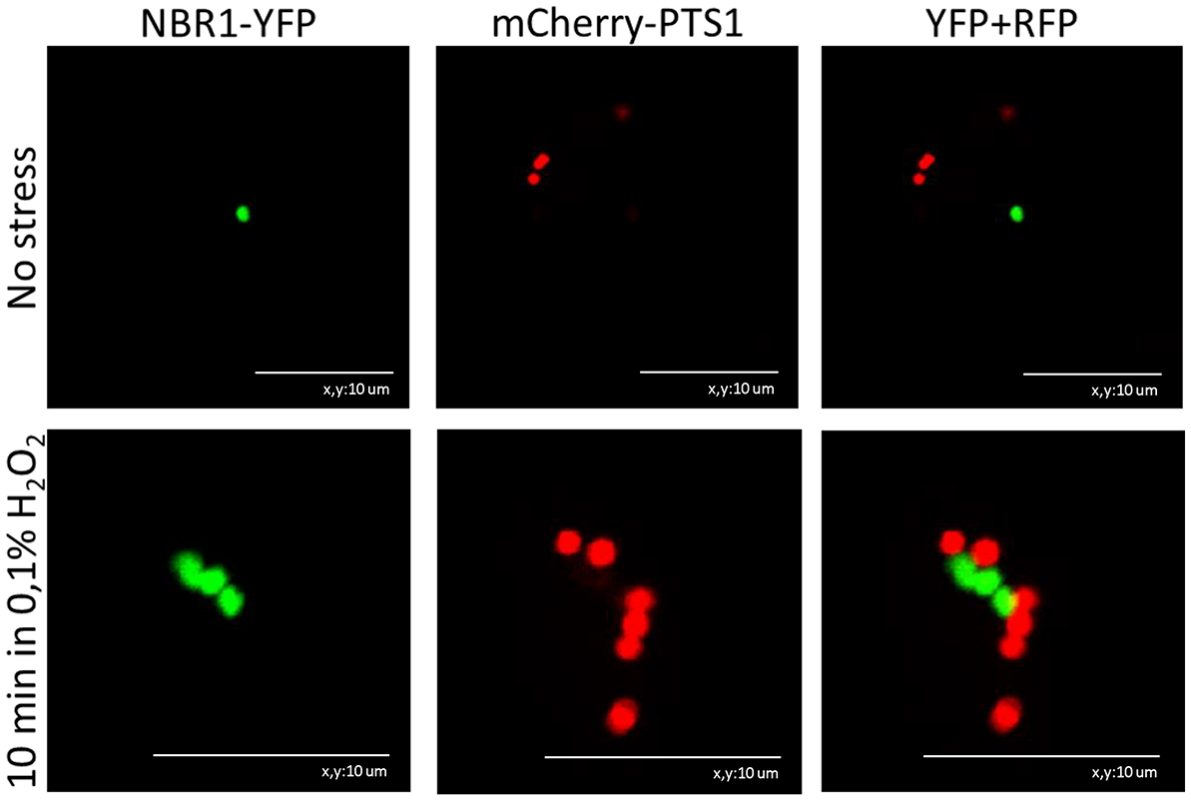
Co-localization of NBR1-YFP with mCherry-PTS1 used as a peroxisomal marker. The markers localize in the proximity of each other mostly after oxidative stress (treatment of the plant tissue with 0.1% H_2_O_2_ for 10 min). The representative micrographs are from *in planta* transient expression experiments. Scale bars, 10 μm.

In mammals, NBR1 is involved in targeting peroxisomes for degradation^28^. Thus, we examined the interaction between *Arabidopsis* NBR1 and peroxisomes. Indeed, after oxidative stress, but not in the control conditions, we observed clustering of transiently co-expressed in *Nicotiana benthamiana* leaves NBR1-YFP and mCherry-PTS used as peroxisomal marker (Fig. 4). This observation and a coordinated movement of both markers (not shown) together with accumulation of peroxisomes in nbr1-KO mutant during stress (Fig. 3) suggests that NBR1 might contribute to remove damaged peroxisomes; however, it is unclear what protein is recognized by NBR1 as a target since several peroxisomal proteins might be implicated in pexophagy^33^. Nevertheless, we failed to detect direct CAT2–NBR1 interactions in the yeast two-hybrid (Y2H) assay using either the low- or the high-copy-number vectors (not shown).

### Oxidative stress reduces photosynthetic pigments contents in all tested lines

**Fig. 5:**
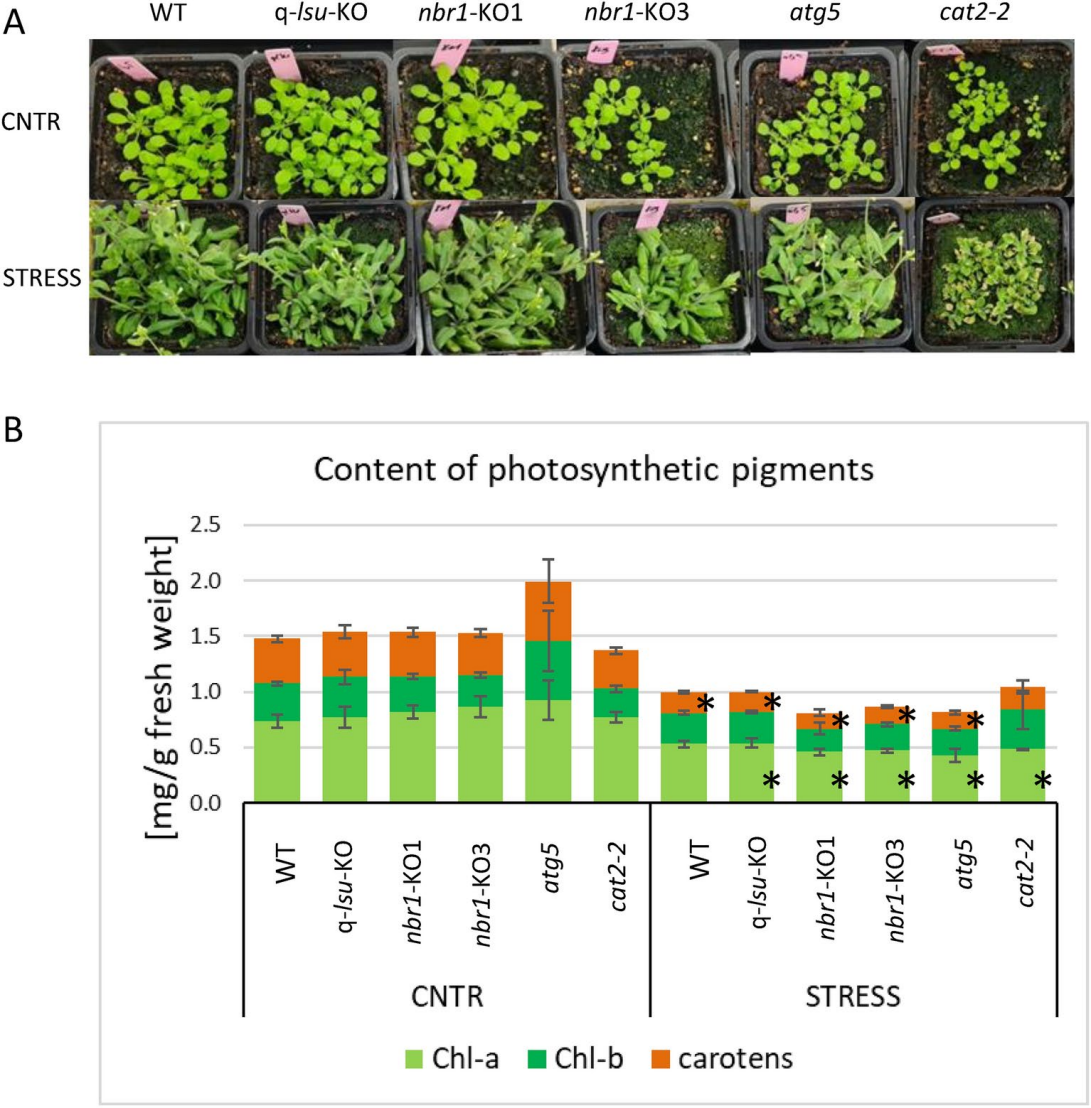
Comparison of oxidative stress tolerance. (**A**) Visualization of soil-grown plants in the absence and presence of oxidative stress (7 day of constant light, 1500 μmol/m^2^/s). (**B**) The chlorophyll *a* (Chl-a), chlorophyll *b* (Chl-b) and carotenoids contents in the analyzed plants. The results shown in (**B**) are from three biological replicates, each with three technical replicates. The differences between the lines grown in the same conditions were statistically insignificant. The statistically significant differences (*p* < 0.05) for each line between the conditions are indicated by asterisks.

Mutations that reduce the function of catalase and peroxisomes increase cellular oxidative stress. This in turn, results in degradation of photosynthetic pigments—chlorophyll *a*, chlorophyll *b*, and carotenoids—which are often used as photo-oxidative markers^34^. We examined the oxidative stress tolerance of the q-*lsu*-KO and *nbr1*-KO mutants. We used two independently obtained *nbr1* deletion mutants, *nbr1*-KO1 and *nbr1*-KO3. In addition, we used the *atg5* mutant defective in autophagy and the *cat2*-2 mutant defective in CAT2 activity. We divided the 14-day-old plants grown in soil into two groups. The control (CNTR) group was maintained for additional 7 days in the same conditions. The second group (STRESS) was subject of a high light stress for 7 days. Exposure to continuous high light stress leads to overproduce chloroplastic ROS that are implicated in both signaling and oxidative damage^35^. Then, we inspected the plants, photographed them, and determine the photosynthetic pigment contents (chlorophyll *a*, chlorophyll *b*, and carotenoids) in their aboveground parts (Fig. 5). After stress, we observed bolting in all but the *cat2-2* mutant, which in contrast to the other lines had a lower mass of green tissues and showed symptoms of premature aging (Fig. 5A). There were no significant differences in the content of individual pigments between the lines grown in the same conditions (CNTR or STRESS; Figs. 4B and S3), however stress reduced the total pigment contents in all lines, particularly the chlorophyll *a* (Chl-a) and carotenoids. According to the rigorous Tukey honestly significant difference (HSD) test, the change in the Chl-a content was significant in all lines except the WT. There was no significant change in the chlorophyll *b* (Chl-b) content after stress in any of the lines, and a significant change in carotenoids contents in all lines except cat2-2 (Figs. 4B and S3). In summary, a role for the selective autophagy cargo receptor NBR1 and autophagy in the plant response to stress could not be proved based on the assay of the photosynthetic pigments content presented in Fig. 3. However, the physiological role of LSU and NBR1 in stress mitigation cannot be excluded and further experiments are needed to investigate the consequences of altered catalases and peroxisome dynamics in the *nbr1* and *lsu* mutants.

### LSU1 directly interacts with CAT2 and CAT3

**Fig. 6:**
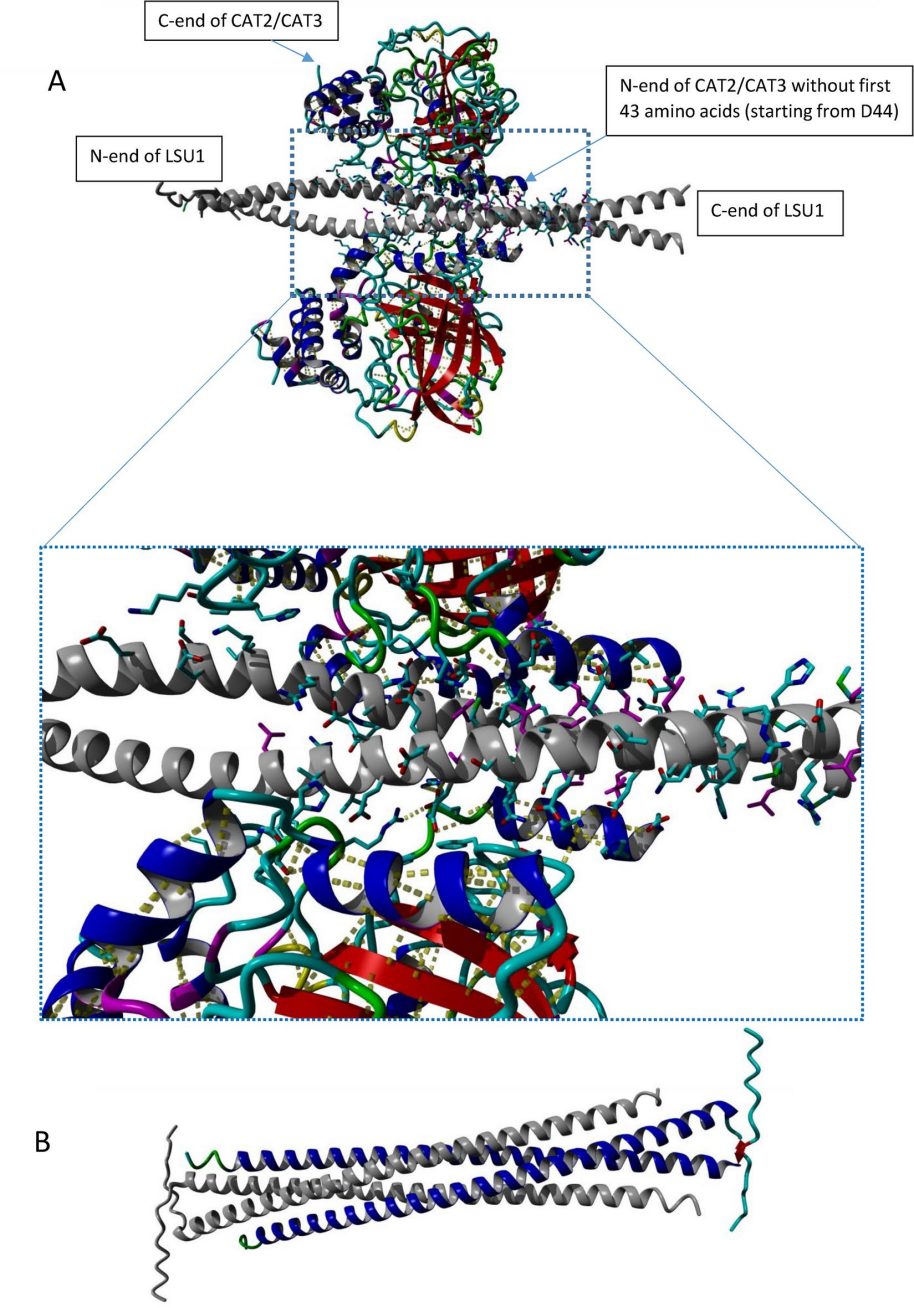
The 3D structure of (**A**) a complex of two CAT2 monomers with an LSU1 dimer and (**B**) an LSU1 tetramer forming a complex of two dimers. According to the 3D model of LSU1 tetramer demonstrated in (**B**), parallel dimers of LSU1 form a tetramer in a nonparallel arrangement.

A direct binding of LSU was previously examined only for CAT2^9^ but it can be expected for other CAT isoforms because all of them have similar structural characteristics^19^. The binding characteristics of CAT2 and CAT3 to LSU1 may differ as suggested by different areas and numbers of YFP-CAT2 and YFP-CAT3 condensates in the q-*lsu*-KO background (Fig. 3). The initial model of the CAT2/CAT3 monomer shows that the N-terminal partially helical fragment is somewhat randomly orientated relative to the globular C-terminal domain. So, the direct application of such a flexible structure in modelling the LSU-CAT complex might be substantially randomly biased. Since the N-terminal part could have been further confirmed experimentally to contribute to the interaction, we decided to analyze separately both fragments interacting with LSU and then make an effort to reconstruct the whole complex. Indeed, the modelling gave a consistent set of structures when the CAT2/CAT3 proteins were divided into two fragments, the N-terminal α-helical peptides (S30-P60), and the N-terminally truncated (E61-I492) proteins. The models revealed two possible regions of interaction in CAT2 and CAT3 monomers (Fig. S4). The first interaction site with LSU1 involves the amino acids in the N-terminal helices (residues 45–58). The second interaction site with LSU1 was refined using the manually created joined model showing two CAT2/CAT3 N-terminally truncated monomers (D44-I492), and the LSU1 dimer. The CAT2/CAT3 residues involved in interaction with LSU1 are located close together in the 3D structural model but they are scattered along the primary sequence (Figs. 6A and S4).

**Fig. 7:**
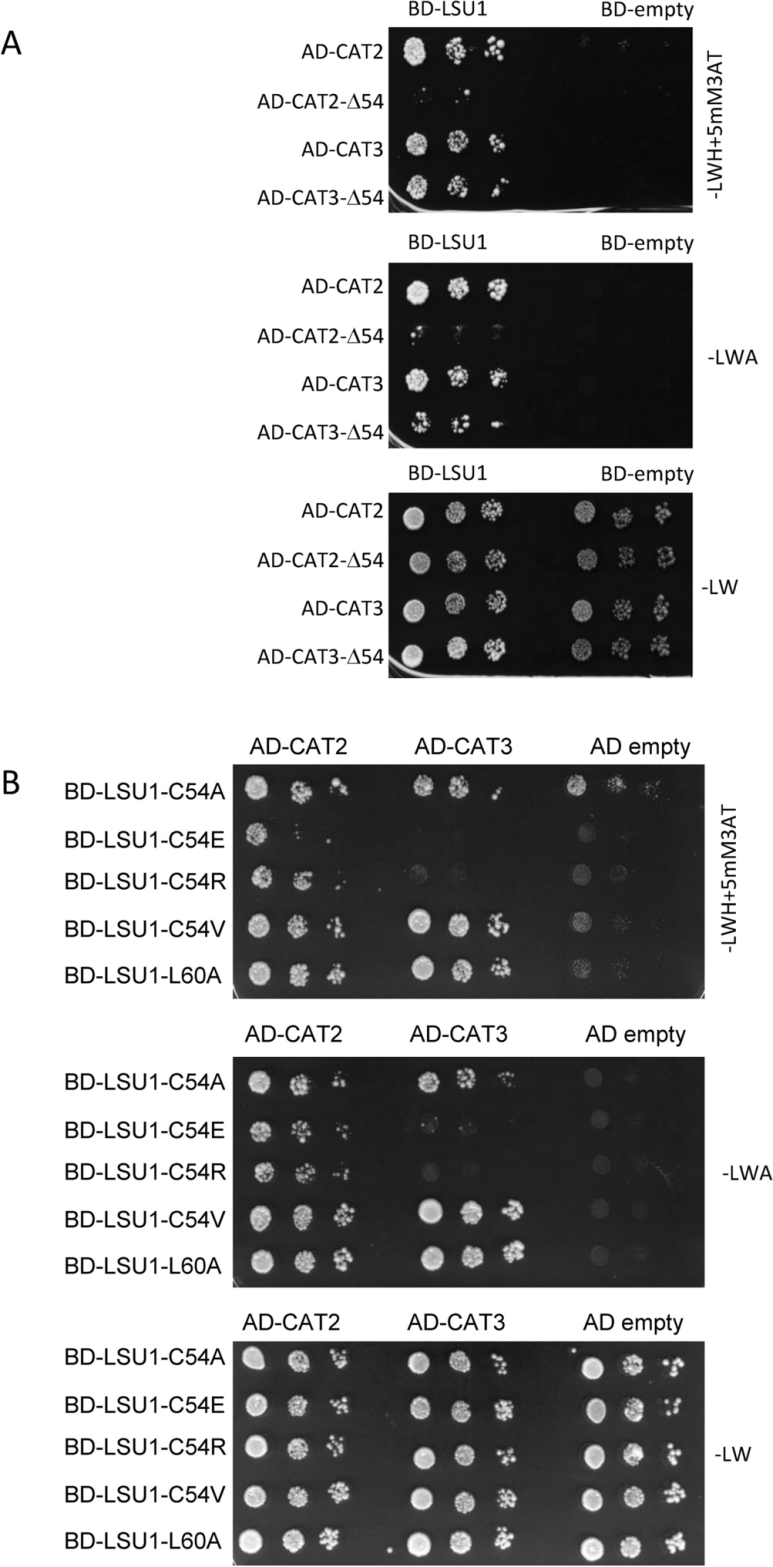
Interaction of CAT2 and CAT3 with LSUs in the Y2H assay. (**A**) The effects of deletion of N-terminal 54 amino acids in CAT2 (AD-CAT2Δ54) and CAT3 (AD-CAT3Δ54) on the interaction with LSU1. (**B**) The interaction between the indicated LSU1 mutants and CAT2 and CAT3. The three dots in each series represent three serial tenfold dilutions of the respective yeast culture from which the same aliquots (5 μL) were spotted on the indicated plates. The growth on –LW medium (lacking leucine and tryptophan) indicates the presence of the pDEST22 and pDEST32 plasmids containing the GAL4 activation (AD) and binding (BD) domains, respectively. The growth on –LWH+5mM3AT medium (lacking histidine in the presence of 5 mM 3-amino-1,2,4- triazole) and –LWA medium (lacking adenine) is possible due to the interaction between the tested proteins.

In an attempt to verify the modelling results, we prepared N-terminally truncated variants of CAT2 and CAT3 and checked their ability to interact with LSU1. Interestingly, the CAT2 variant with deletion of 54 N-terminal amino acids presented a markedly reduced interaction with LSU1, while the CAT3 variant with the identical deletion had only a slightly reduced interaction with LSU1 (Fig. 7A). We also evaluated the CAT2 and CAT3 interaction with mutated variants of LSU1: C54A, C54E, C54R, C54V, and L60A. Consistent with previous observations, the C54E and C54R mutations had the greatest impact on weakening the LSU1–CAT2 interaction^9^. The interaction between CAT3 and these two LSU1 mutants was almost completely abolished (Fig. 7B). These results confirms that the binding characteristics of CAT2 and CAT3 to LSU1 are indeed different. However, additional experiments are needed for precise mapping of the contact sites.

Molecular modelling has pointed out that the N-terminal flexible fragment and the C-terminal truncated protein may independently interact with LSU. Following this result, we demonstrated experimentally that the N-terminal fragment is undoubtedly involved in LSU binding, discriminating CAT2 and CAT3. However, it must be noted that this fragment is also packed intensely in the tetramer structure. Consequently, any rational mutation analysis must involve studies on putative CAT2/3 oligomeric forms. So, purely biophysical methods, namely MS-HDX or NMR perturbation approach, seem to be the best method to map interactions, the thermodynamics of which could be further studied with MST or ITC. However, such an approach substantially redefines the problem, which remains interesting and worth further investigation.

The 3D models of the CAT2/3-LSU1 dimer complexes suggest that the N-terminus of CAT2/3 is directed towards the C-termini of LSU1-LSU1 dimer. Therefore, the geometric properties of LSU1-LSU1 coiled coil might jeopardize the formation of an active GAL4, since the CAT2, CAT3 and LSU1 proteins used in Y2H assays have the domains of GAL4 localized on their N-ends. In search of the explanation of this discrepancy between the model and experimental Y2H results we examined the possibility of antiparallel dimer formation by LSU1. The modeling revealed that LSU1 can form antiparallel complexes of parallel dimers (Fig. 6B). This model shows the most probable formation of LSU multimers and it highlights also the possible role of C54 in multimer formation (not shown).

## Discussion

In this study, two distinct stressors were employed to highlight the roles of LSU and NBR1 in stress mitigation. A common characteristic of the used stressors is their ability to induce an internal burst of reactive oxygen species (ROS)^36^. An adequate response to environmental changes and mitigation of harmful ROS in optimal and stress conditions are critical for appropriate plant growth and development. Catalases are important enzymes involved in removal of H_2_O_2_. They are present in different cellular compartments, especially peroxisomes, which are the main sites of H_2_O_2_ generation in plant cells. Numerous mechanisms in plant cells contribute to the precise control of catalase activity. Moreover, the distribution and number of peroxisomes are critical for plants. However, the maturation pathway of plant catalases has been poorly characterized^37^. The amount of insoluble catalase increases in the *nbr1* mutant during heat stress, suggesting involvement of NBR1 in catalase degradation upon the heat stress^38^. Our results indicate that autophagy is involved in catalase degradation because the *atg5* mutant defective in autophagy accumulated catalase (Fig. 1). In the same series of experiments, we failed to detect a significant difference in catalase activity in the q-*lsu*-KO and *nbr1*-KO1 mutants and the lines that overexpress LSU1 or NBR1 compared with WT. We also demonstrated that YFP-CAT2 and YFP-CAT3 formed smaller condensates in the q-*lsu*-KO and *nbr1*-KO1 mutants (Fig. 2). This observation, in turn, suggests that both proteins are needed to form complex protein structures that include catalases. They could be involved either in their degradation, maturation (tetramer formation), or intracellular trafficking. The “YFP cleavage” experiment paradoxically showed stronger degradation of CAT2 in the *nbr1*-KO line (Fig. S2). This may suggest a hypothesis for future verificationthat the rate of degradation correlates negatively with the area of the condensates formed by CAT2 and CAT3. These phenomena need to be elucidated further.

Peroxisomes in *Arabidopsis* proliferate in response to a variety of biotic and abiotic stresses^39^. The large aggregates (presumably peroxisomes) we observed with N-BODIPY in the q-*lsu*-KO, *nbr1*-KO1, and *atg5* mutants after osmotic stress (Fig. 3) support the hypothesis that LSUs and NBR1 are crucial for the removal of the peroxisomes damaged by stress. Both proteins are probably indirectly involved in mitigation of oxidative stress in plants by influencing the homeostasis of the ROS mitigating enzymes, such as intracellular transport, aggregation or recycling during stress.

The photosynthetic pigments chlorophyll *a*, chlorophyll *b*, and carotenoids are often used as photo-oxidative markers^34^. Therefore, we decided to assay their contents in the plants grown in the control conditions and exposed to high light stress. Interestingly, we did not observe significant differences between WT, q-*lsu*-KO, *nbr1*-KO1, *nbr1*-KO3, and *atg5* (Figs. 4 and S3). It is plausible that our stress conditions were too mild to observe differences.

We also observed co-localization of NBR1-YFP with mCherry-PTS1 used as a peroxisomal marker and direct binding of *Arabidopsis* catalases, CAT2 and CAT3 with LSU1 (Figs. 5 and 7). The structural models of all three CAT isoforms from *A. thaliana* have been reported previously^19^. In the present study, we created structural models of the LSU1–CAT2/CAT3 complexes (Fig. 6). According to these models, catalases interact with LSU1 via their N-terminus and a second region of interaction. Because the N-terminus of catalases forms an extended arm that participates in the formation of the tetramer^40^, it is tempting to speculate that the possible interaction with LSU1 might influence the formation of CAT tetramers, but it is only hypothesis and would require further experimental support. Interestingly, N-terminal fragment of *Arabidopsis* CAT2 was identified as an accelerator of jasmonic acid biosynthesis by interacting with and promoting the activity of peroxisomal ACX2/3, an enzyme involved in jasmonic acid biosynthesis^41^. LSU might modulate such functions by competing about the access to CAT N-terminus.

Under normal growth conditions, cells produce low levels of reactive oxygen species (ROS). However, under biotic and abiotic stress, ROS production increases, which can impair various cellular functions by damaging nucleic acids, oxidizing proteins, and causing lipid peroxidation^42^. Plants have developed robust defense mechanisms against ROS, utilizing both enzymatic and non-enzymatic processes. Superoxide dismutases and catalases play key roles in mitigating ROS, supporting plant metabolism, defense, and signal transduction. Studies have shown that LSU1 physically interacts with iron-type superoxide dismutase FSD2 and can enhance its enzymatic activity^15^. Our research contributes to the growing evidence linking LSU proteins to stress responses by identifying catalases as partners of LSU proteins and suggesting that LSU may influence intracellular CAT distribution. Considering the moderate co-localization of LSU1 with peroxisomes (Fig. S6) and the strong interaction between LSU1 and catalases, it is quite probable that LSU1–catalase interactions occur in the cytosol rather than in peroxisomes.

The role of NBR1 in the processes investigated in this study might be explained by its involvement as a selective autophagy cargo receptor in response to environmental stresses, particularly stresses resulting in protein damage^43^. Although in animals NBR1 functions as pexophagy receptor, in plants autophagic degradation of peroxisomes is rather NBR1-independent^43^, however implication of NBR1 in heavy metal cadmium-induced, reactive oxygen species-dependent pexophagy in Arabidopsis was also reported^44^.

In summary, we postulate that LSU1 and NBR1 contribute to the control of catalase and/or peroxisome homeostasis in plants. It is plausible that both proteins might be involved in formation of the aggregates by inactive or defective subunits of catalase facilitating their autophagic degradation.

## Methods

### Gene cloning, oligonucleotides, and vectors

The genetic constructs, complementary DNA (cDNA) fragments, and oligonucleotides used in this work are listed in Table S1. Most of the cDNA inserts were obtained by polymerase chain reaction (PCR) amplification. The plasmids were constructed either via conventional restriction digestion/ligation or recombined into the respective destination vectors by Gateway™ technology^45^. The following Gateway™ destination vectors were used: pDEST22 and pDEST32 as low-copy vectors for Y2H experiments, and pH7WGY2 for obtaining fusions with N-terminally located YFP^46^.

### *A. thaliana* plants, transformation, and growth conditions

All described experiments were performed using WT *A. thaliana* of the Columbia (Col-0) ecotype and its derivatives: *nbr1*-KO1, NBR1-OX7.5, NBR1-YFP^47^, *q-lsu*-KO^6^, *atg5*-1 (NASC stock nr: N39993), *cat2*-2 (NASC stock nr: N557998)^48^, and LSU1-YFP (lab stock). Transgenic lines generated for the purposes of this work were obtained by *Agrobacterium*-mediated genetic transformation of WT using the floral dip method^49^ with plasmids encoding NBR1-YFP, YFP-CAT2, and YFP-CAT3, each under the control of the cauliflower mosaic virus 35S promoter. To test oxidative stress tolerance, the plants were grown in soil for 14 days and then exposed to oxidative stress (7 days of constant light, 1500 μmol/m^2^/s) or maintained for the same amount of time in the control conditions. For other experiments, plants were cultivated in vitro. Seeds were dry sterilized as described previously^50^ and sown onto half-strength Hoagland’s medium containing 0.8% agar, and stratified at 8 °C for 2 days. Plates were placed vertically in a growth chamber with a photoperiod of 8 h of light and 16 h of darkness at 19/21 °C. Fourteen-day-old plants were used for western blotting, peroxisome quantification using N-BODIPY, quantitative assessment of YFP-CAT2 and YFP-CAT3 condensates across different lines, and for autophagy induction before the “YFP-cleavage” assay.

### Transient expression in *N. benthamiana* leaves

The leaves of soil-grown 7-week-old *N. benthamiana* plants were infiltrated with *Agrobacterium tumefaciens* cells (strain GV3101) as described previously^51^ using the combinations of the plasmids indicated in the respective figures. After agroinfiltration, plants were grown in the greenhouse for 3 days; then, the leaves were used for confocal fluorescence microscopy.

### Western blotting

Plant material was homogenized in 40 μL of extraction buffer (50 mM Tris‐HCl, pH 8.0, 0.5 M NaCl, 8 M urea, and 1% Triton X-100) with 1 μL of Protein Inhibitor Cocktail (Merck) and centrifuged (13,000 rpm, 4 °C, 10 min). Proteins from the supernatant were separated on 12% SDS‐PAGE (sodium dodecyl sulfate–polyacrylamide gel electrophoresis) gels, transferred to the nitrocellulose membrane (BioRad, Hercules, CA, USA), and visualized by staining with Ponceau S (loading control). Membranes were incubated with 5% nonfat dry milk to block nonspecific protein binding and then probed with the primary antibody, monoclonal mouse anti‐GFP IgG (Roche, Mannheim, Germany), or polyclonal rabbit anti-CAT (catalase peroxisomal marker, Agrisera, Vannas, Sweden) diluted 1:1000 in PBS (Phosphate Buffered Saline) for 1 h at room temperature. Then, the membrane was washed in PBS and incubated with the secondary antibody (goat anti-mouse or anti-rabbit IgG [whole molecule] conjugated to alkaline phosphatase; Sigma-Aldrich, St. Luis, MI, USA) diluted 1:10000 in PBS for 1 h at room temperature. Protein bands were visualized by adding 5 mL of 1-Step NBT/BCIP Substrate Solution (Thermo Scientific, Waltham, MA, USA) at room temperature. For semi-quantitative analysis, the respective bands were quantified after scanning using the ImageJ software^52^. The intensity of the bands on the Westernblot membrane was normalized to the amount of the Rubisco protein on the controlled stained gel. The experiments were performed in triplicate; the mean/representative data are shown in the figures.

### YFP-cleavage assay

Fourteen-day-old plants grown in vitro in a Petri dish, as described above, were transferred for 36 h to a liquid layer of medium with 1 µm AZD8055 (an autophagy inducer) or without it (control) and incubated in a fitotron chamber. Protein was isolated and western blotting was performed as described above. Equal amounts of total protein were loaded onto the gel (40 µg per well). The membranes were scanned and a semi-quantitative analysis of the ratio of the YFP-CAT2 fusion protein level to the free YFP- fraction was performed. The immunostained bands were normalized to the Rubisco band visualized by Ponceau S staining for each blot. Membranes were analyzed and quantified using ImageJ software^52^.

### Catalase activity assay

Protein extracts from seedlings were prepared according to Yoshimoto et al.^53^. Briefly, 15 seedlings were homogenized on ice in a buffer containing 100 mM Tris–HCl pH 8.0, 20% glycerol, and 30 mM DTT (dithiothreitol), and then centrifuged at 17,750 g for 15 min. The resulting supernatants were used to measure catalase activity spectrophotometrically in 50 mM KH_2_PO_4_ buffer, by monitoring the consumption of H_2_O_2_ at 240 nm over time at room temperature. Catalase activity was normalized to the total protein level in the extract determined by the Bradford method^54^ or to the protein level of catalase determined by immunoblotting analysis using an anti-catalase antibody.

### Confocal fluorescent microscopy

Observations were made using a Nikon Eclipse TE2000-E inverted confocal microscope (Nikon Corporation, Tokyo, Japan). For YFP visualization, a 488 nm laser (Sapphire 488–20 CDRH; Coherent Inc., Saxonburg, USA) and a 515/30 filter were used. For mCherry, a 543 nm laser (helium–neon laser; Melles Griot, Albuquerque, NM, USA), and a 605/75 filter were used. The same parameters as for YFP staining were used for N-BODIPY peroxisome staining^31^. Image data were analyzed using EZ-C1 3.90 FreeViewer (Nikon Corporation) and the ImageJ software^52^.

### Quantitative assessment of the YFP-CAT2 and YFP-CAT3 condensates

Individual images of the CAT2 and CAT3 mutants (WT, *nbr1*-KO1, and q-*lsu*-KO) were acquired as described above. Images were taken in the form of a Z-stack with a depth of 50 μm from the surface of the specimen, with 2-μm steps. Images were prepared for processing using EZ-C1 3.90 FreeViewer. Subsequently, three smaller images were extracted from each original image of individual lines and scaled to a size of 150 × 150 pixels using the Gimp2 software (ver. 10.36). Selected images were analyzed using the ImageJ software^52^. The procedure involved transforming the image into an 8-bit format, applying a threshold, and setting the binary-watershed parameter. After image analysis and setting the outlining counting parameter, the program generated a new image displaying the number of points in the analyzed area and the surface area of each region. Statistical analysis for each area was performed using the Statistica software (13.3, TIBCO Software, Palo Alto, CA, USA).

### Peroxisome quantification using N-BODIPY

To induce osmotic stress, 14-day-old *Arabidopsis* plants were flooded with 0.3 M NaCl solution (treatment) or water (control) on Peri dishes in which they grew for 3 h. Then, the plants were washed, stained for 15 min with N-BODIPY solution (1 µM 8-(4-nitrophenyl) Bodipy in water), and rinsed with water to wash off the excess of N-BODIPY before observation. The stained and washed plants were mounted on a microscope slide, and the roots were observed. Pictures were captured and analyzed quantitatively as described in the “Quantitative assessment of YFP-CAT2 and YFP-CAT3 condensates” section.

### Chlorophyll and carotenoid contents

Entire seedlings were collected and chlorophyll and carotenoids contents were measured as described previously^55^.

### Molecular modeling

3D models of the CAT2–LSU1 and CAT3–LSU1 complexes were constructed using locally installed AlphaFold2^56^. Usually, 25 models were obtained, of which the 5–6 with the highest scores were analyzed. Inspection of the models, including visualization and contact analysis, and further modelling was done with the Yasara software^57^. The joined model showing two CAT2 monomers, and the LSU1 dimer was manually created. First, the model was preoptimized by 3 ns simulated annealing at 100 K. The second CAT2 monomer was added by symmetry based on the model of the first CAT2 molecule. The entire system was optimized by 5 ns molecular dynamics simulation at a temperature increasing from 100 K to room temperature.

### Y2H experiment

Y2H analysis was performed in Y2HGold (Takara Bio USA, Inc, San Jose, CA, USA). *Saccharomyces cerevisiae* strains were transformed with the pDEST22 and pDEST32 plasmids containing the GAL4 activation domain (AD) and binding domain (BD), respectively, according to standard procedures. The yeasts were plated and selected on synthetic media lacking leucine and tryptophan (− LW), and protein–protein interactions were evaluated on media without histidine in the presence of 3-amino-1,2,4- triazole (–LWH+3-AT) at 5 mM, or without adenine (− LWA). The plates were incubated at 30 °C for 3–4 days.

### Statistical analysis

The differences between the probes were analyzed usually using the Statistica 12 software using one way ANOVA, Tukey HSD test. Differences were marked as significant at *p* < 0.05. The t-test was used to analyze the results presented in Fig. 2 as indicated in the respective legend.

## Supplementary Information

Supplementary Information.

## Data Availability

All data generated or analyzed during this study are included in this published article and its supplementary information files. Arabidopsis strains used in this study, including the ecotype Col-0 and the insertion mutants atg5-1 (NASC stock nr: N39993) and cat2-2 (NASC stock nr: N557998) are available upon request at the Arabidopsis Biological Resource Center (https://abrc.osu.edu/). The other deletion and overexpression lines described in this study (nbr1-KO1, NBR1-OX7.5, NBR1-YFP, q-lsu-KO, and LSU1-YFP) are available free upon request from the Plant Protein Homeostasis Laboratory stocks at IBB PAS by contacting corresponding author or institution. Plasmids used are also available upon request from the Plant Protein Homeostasis Laboratory stocks. Additional data supporting the findings of this study are available from the corresponding authors (A.S. or M.O.) upon request.
