# Supplementary material for: LSU family members and NBR1 are novel factors that contribute to homeostasis of catalases and peroxisomes in Arabidopsis thaliana
Source: Sci Rep. 2024 Oct 25;14:25412. doi: 10.1038/s41598-024-76862-4 (PMC11511919; doi:10.1038/s41598-024-76862-4)
Supplement: Supplementary file 1 — Supplementary Information. [file 41598_2024_76862_MOESM1_ESM.pdf]

Supplementary material

Suppl. Table 1 List of plasmids and oligonucleotides used in this work.

**Plasmids**		
**Plasmid name**	**Gene/AGI number/description**	**Refrence/source**
LSU1-YFP<br>BD-LSU1	*LSU1*/ At3g49580	Niemiro-et. al 2020<br>Niemiro-et. al 2020
BD-LSU2	*LSU2*/ At5g24660	Niemiro-et. al 2020
BD-LSU3	*LSU3*/ At3g49570	Niemiro-et. al 2020
BD-LSU4	*LSU4*/ At5g24655	Niemiro-et. al 2020
LSU1_L60A<br>LSU1_C54A<br>LSU1_C54E<br>LSU1_C54R<br>LSU1_C54V	Mutated *LSU1* variants	Niemiro-et. al 2020<br>Niemiro-et. al 2020<br>Niemiro-et. al 2020<br>Niemiro-et. al 2020<br>This work
NBR1-YFP	*NBR1*/ At4g24690	Tarnowski et al. 2020
AD-CAT2<br>YFP-CAT2	*CAT2*/ At4g35090	This work
AD-CAT3<br>YFP-CAT3	*CAT3*/ At1g20620	This work
AD-CAT2-Δ54<br>AD-CAT3-Δ54	*CAT2* or *CAT3*with deletions removing sequence encoding 54 N-terminal residues	This work
mCherry-PTS (px-rk CD3-983)	peroxisome targeting signal (PTS) marker	Nelson et al. 2017

**Oligonucleotides for PCR**		
**Oligonucleotide sequence (5'-3'); forward and reverse**	**Gene**	**Product size**
CACCATGGATCCTTACAAGTAT<br>TTAGATGCTTGGTCTCACGTTC	CAT2/ At4g35090	1479
CACCATGGACAGGGAACGGATTCCAGAGC<br>TTAGATGCTTGGTCTCACGTTC		1324
CACCATGGATCCTTACAAGTAT<br>TTAGATGCTTGGCCTCACGTTC	CAT3/ At1g20620	1479
CACCATGACCAGAGAGAGGATCCCTGAG<br>TTAGATGCTTGGCCTCACGTTC		1324

References

Nelson, Brook K. et al. A multicolored set of in vivo organelle markers for co-localization studies in Arabidopsis and other plants. Plant J 51.6, 1126-1136, doi: 10.1111/j.1365-313X.2007.03212.x (2017)

Niemiro, A. et al. Similar but Not Identical-Binding Properties of LSU (Response to Low Sulfur) Proteins From Arabidopsis thaliana. Front Plant Sci 11, 1246, doi:10.3389/fpls.2020.01246 (2020).

Tarnowski, L. et al. A selective autophagy cargo receptor NBR1 modulates abscisic acid signalling in Arabidopsis thaliana. Sci Rep 10, 7778, doi:10.1038/s41598-020-64765-z (2020).

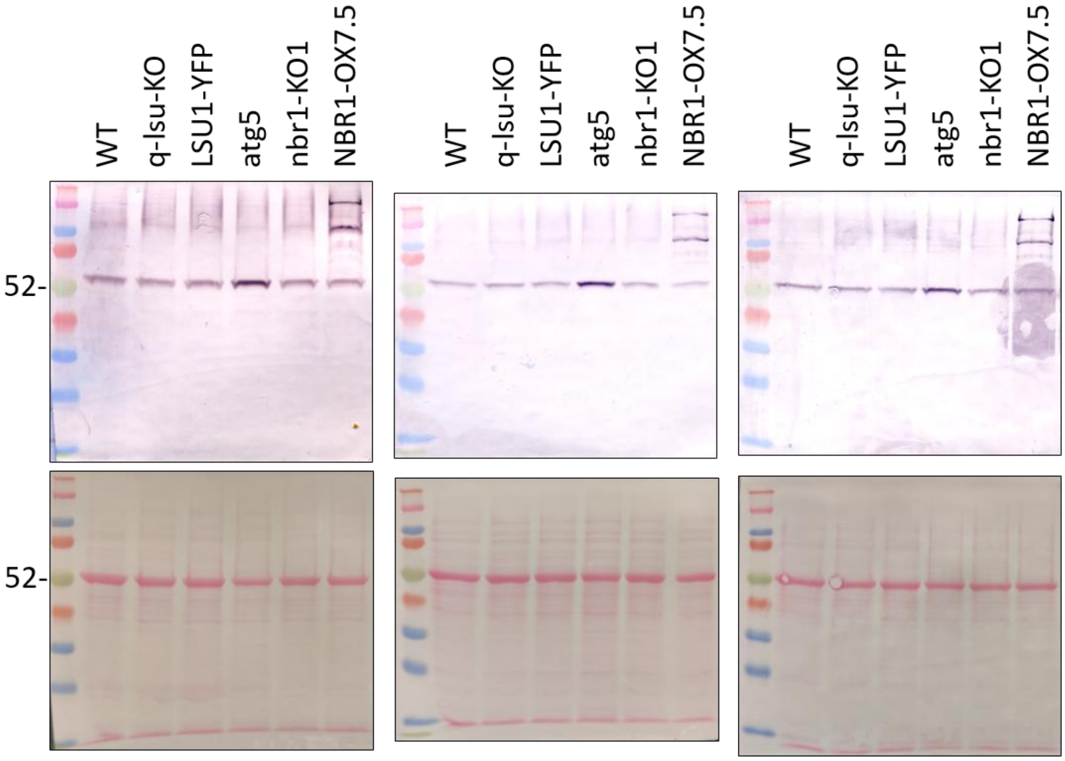

Fig. S1. Biological repetitions of western blots for monitoring the amount and activity of catalase. Western blotting of total plant extracts. In NBR1-OX, the anti-catalase antibody detected two bands: the expected catalase band at 52 kDa and a chimeric NBR1–TAP fusion proteins (the TAP-tag includes a fragment of an immunoglobulin heavy chain, causing some non-specific binding by the anti-catalase antibody). Ponceau S staining (protein loading control) confirmed equal protein loading across lanes based on Rubisco small subunit abundance. Spectr Multicolor Broad Range Protein Ladder (Thermo Fisher Sci.) was used as a marker.

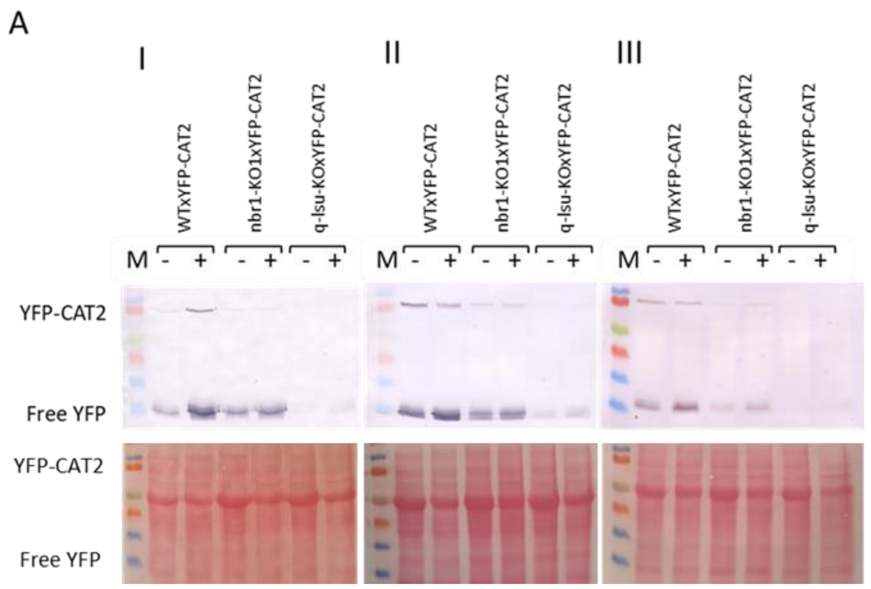

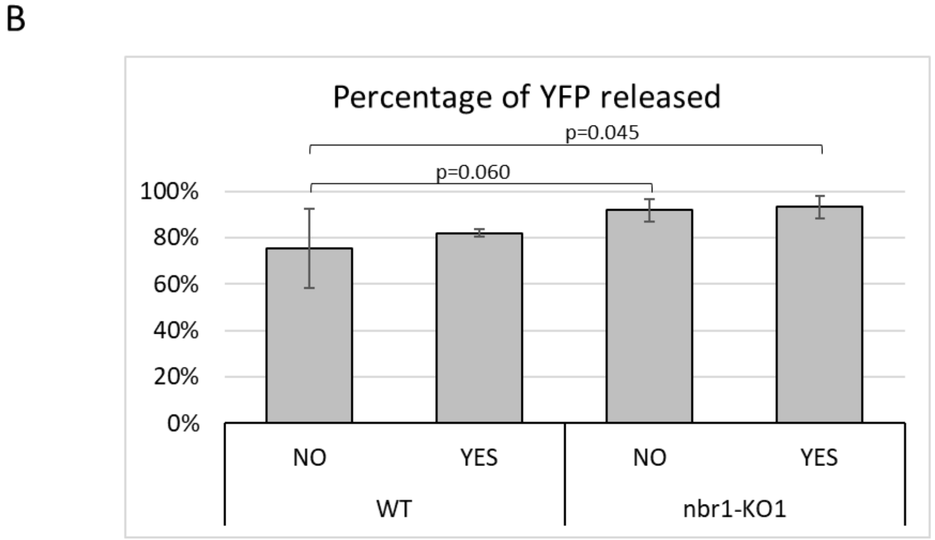

Fig. S2. YFP-cleavage assay

(A) “YFP-cleavage” western blot membranes: Representative images of “YFP cleavage” assay membranes from three biological replicates of YFP-CAT2 in WT, q-lsu-KO and nbr1-KO1 background are shown. Each membrane displays the distinct bands corresponding to the YFP-CAT2 protein and free YFP, indicating the efficiency of the cleavage reaction. Control plants were treated with water, while the other plants were exposed to an 1 µM AZD8055 solution for 36 hours. The lower part shows the membranes after protein transfer stained with Ponceau S, used for Western blot analysis shown in the upper part. All plants were grown on [nS] medium under short-day in optimal conditions. M is the molecular weight marker, - and + mean the absence and presence of AZD8055 treatment, respectively. Spectr Multicolor Broad Range Protein Ladder (Thermo Fisher Sci.) was used as a marker.

(B) Quantitative analysis of “YFP cleavage” test: The graph presents the percentage of YFP released (%), averaged across the three biological replicates. The results were normalized to the Rbc band visualized by Ponceau S staining. Error bars represent the standard deviation (SD) from three independent experiments. Statistical significance was determined using posthoc LSD test in Statistica software. The differences are marked with the p values indicated. NO and YES mean the absence and presence of AZD8055 treatment, respectively. The q-lsu-KO line was not analysed quantitatively because of the low level of the protein detected on Westernblot.

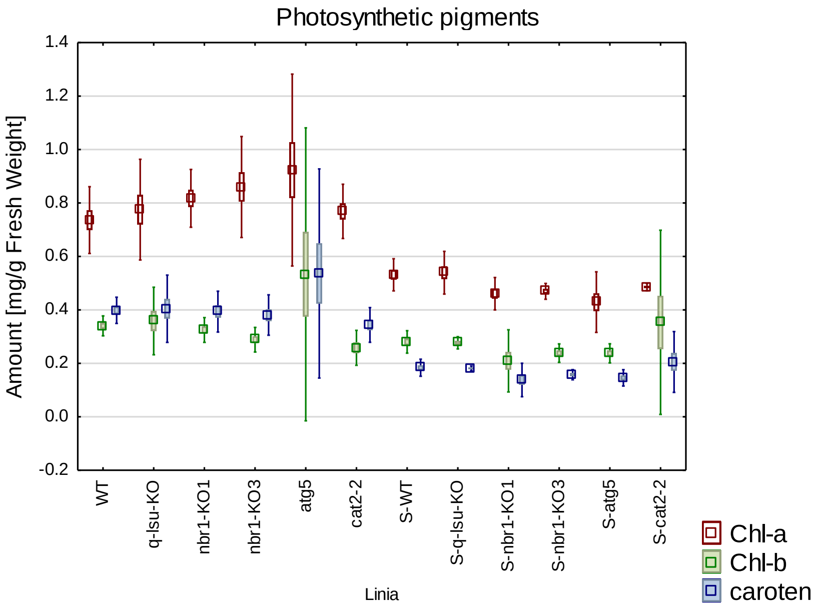

	Tukey HSD test; Variable: Chl-a (stress-swietlny-do stat)Marked differences are significant at p < .05000											
Linia	{1}	{3}	{4}	{5}	{2}	{6}	{7}	{8}	{9}	{10}	{11}	{12}
WT {1}		0.999938	0.964824	0.666266	0.145291	0.999989	0.081133	0.105026	0.005977	0.008367	0.001778	0.015383
q-lsu-KO {3}	0.999938		0.999856	0.952972	0.418143	1.000000	0.020147	0.026879	0.001359	0.001879	0.000458	0.003450
nbr1-KO1 {4}	0.964824	0.999856		0.999853	0.831442	0.999470	0.003974	0.005393	0.000340	0.000432	0.000191	0.000719
nbr1-KO3 {5}	0.666266	0.952972	0.999853		0.994615	0.926219	0.000816	0.001071	0.000172	0.000187	0.000149	0.000233
atg5 {2}	0.145291	0.418143	0.831442	0.994615		0.360946	0.000184	0.000202	0.000144	0.000145	0.000143	0.000147
cat2-2 {6}	0.999989	1.000000	0.999470	0.926219	0.360946		0.025452	0.033841	0.001715	0.002382	0.000556	0.004400
S-WT {7}	0.081133	0.020147	0.003974	0.000816	0.000184	0.025452		1.000000	0.987016	0.995466	0.857546	0.999683
S-q-lsu-KO {8}	0.105026	0.026879	0.005393	0.001071	0.000202	0.033841	1.000000		0.972144	0.988334	0.795520	0.998686
S-nbr1-KO1 {9}	0.005977	0.001359	0.000340	0.000172	0.000144	0.001715	0.987016	0.972144		1.000000	0.999992	0.999999
S-nbr1-KO3 {10}	0.008367	0.001879	0.000432	0.000187	0.000145	0.002382	0.995466	0.988334	1.000000		0.999910	1.000000
S-atg5 {11}	0.001778	0.000458	0.000191	0.000149	0.000143	0.000556	0.857546	0.795520	0.999992	0.999910		0.998006
S-cat2-2 {12}	0.015383	0.003450	0.000719	0.000233	0.000147	0.004400	0.999683	0.998686	0.999999	1.000000	0.998006	

	Tukey HSD test; Variable: Chl-b (stress-swietlny-do stat)Marked differences are significant at p < .05000											
Linia	{1}	{2}	{3}	{4}	{5}	{6}	{7}	{8}	{9}	{10}	{11}	{12}
WT {1}		0.439535	1.000000	1.000000	0.999931	0.995400	0.999715	0.999530	0.880161	0.975313	0.973882	1.000000
atg5 {2}	0.439535		0.579011	0.333993	0.153667	0.072917	0.126410	0.116655	0.019234	0.042991	0.042053	0.539918
q-lsu-KO {	1.000000	0.579011		0.999999	0.998833	0.978115	0.996904	0.995608	0.770961	0.926829	0.923787	1.000000
nbr1-KO1 {	1.000000	0.333993	0.999999		0.999998	0.999259	0.999984	0.999968	0.942864	0.992900	0.992350	1.000000
nbr1-KO3 {	0.999931	0.153667	0.998833	0.999998		1.000000	1.000000	1.000000	0.996666	0.999951	0.999943	0.999402
cat2-2 {6}	0.995400	0.072917	0.978115	0.999259	1.000000		1.000000	1.000000	0.999962	1.000000	1.000000	0.984989
S-WT {7}	0.999715	0.126410	0.996904	0.999984	1.000000	1.000000		1.000000	0.998727	0.999992	0.999990	0.998263
S-q-lsu-KO	0.999530	0.116655	0.995608	0.999968	1.000000	1.000000	1.000000		0.999171	0.999997	0.999996	0.997427
S-nbr1-KO	0.880161	0.019234	0.770961	0.942864	0.996666	0.999962	0.998727	0.999171		1.000000	1.000000	0.804237
S-nbr1-KO	0.975313	0.042991	0.926829	0.992900	0.999951	1.000000	0.999992	0.999997	1.000000		1.000000	0.943798
S-atg5 {11	0.973882	0.042053	0.923787	0.992350	0.999943	1.000000	0.999990	0.999996	1.000000	1.000000		0.941237
S-cat2-2 {1	1.000000	0.539918	1.000000	1.000000	0.999402	0.984989	0.998263	0.997427	0.804237	0.943798	0.941237	

	Tukey HSD test; Variable: caroten (stress-swietlny-do stat)Marked differences are significant at p < .05000											
Linia	{1}	{3}	{4}	{5}	{2}	{6}	{7}	{8}	{9}	{10}	{11}	{12}
WT {1}		1.000000	1.000000	1.000000	0.343838	0.995583	0.020349	0.018590	0.002786	0.006582	0.003930	0.049711
q-lsu-KO {	1.000000		1.000000	0.999999	0.405023	0.989657	0.015720	0.014349	0.002149	0.005061	0.003018	0.038884
nbr1-KO1 {	1.000000	1.000000		1.000000	0.298399	0.998027	0.025030	0.022888	0.003446	0.008160	0.004881	0.060449
nbr1-KO3 {	1.000000	0.999999	1.000000		0.199940	0.999873	0.042418	0.038892	0.006051	0.014158	0.008546	0.098833
atg5 {2}	0.343838	0.405023	0.298399	0.199940		0.051088	0.000171	0.000168	0.000145	0.000150	0.000146	0.000230
cat2-2 {6}	0.995583	0.989657	0.998027	0.999873	0.051088		0.171160	0.159050	0.029436	0.065137	0.040780	0.337312
S-WT {7}	0.020349	0.015720	0.025030	0.042418	0.000171	0.171160		1.000000	0.999047	0.999996	0.999837	0.999999
S-q-lsu-KO	0.018590	0.014349	0.022888	0.038892	0.000168	0.159050	1.000000		0.999381	0.999998	0.999907	0.999999
S-nbr1-KO	0.002786	0.002149	0.003446	0.006051	0.000145	0.029436	0.999047	0.999381		1.000000	1.000000	0.977188
S-nbr1-KO	0.006582	0.005061	0.008160	0.014158	0.000150	0.065137	0.999996	0.999998	1.000000		1.000000	0.998609
S-atg5 {11	0.003930	0.003018	0.004881	0.008546	0.000146	0.040780	0.999837	0.999907	1.000000	1.000000		0.991120
S-cat2-2 {1	0.049711	0.038884	0.060449	0.098833	0.000230	0.337312	0.999999	0.999999	0.977188	0.998609	0.991120	

Fig. S3. The statistical data from the analysis of the photosynthetic pigments contents in plants grown in soil and exposed to the high light stress shown in Figure 5.

A

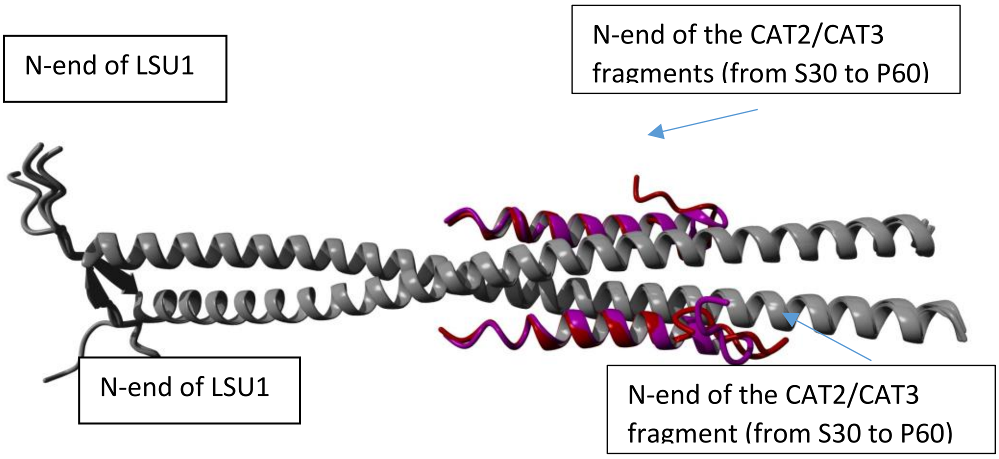

B

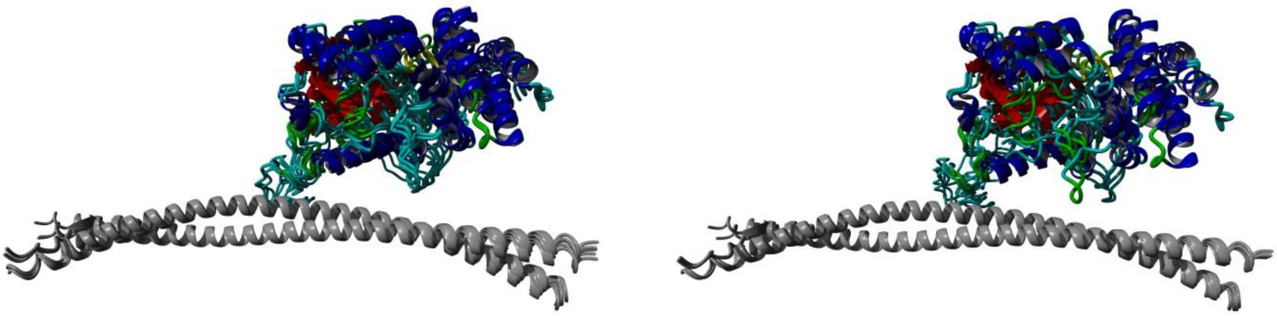

Fig. S4. The molecular models of the structure of the complexes of CAT2/CAT3 fragments with LSU1 dimer. The S30-P60 fragments of CAT2/CAT3 (A) and the Δ1-60 fragments of CAT2/CAT3 (B) were used for the modeling of the complex structure.

A

```
                            |         |         |         |         |         |         |         |         |        100
CAT1        MDPYRVRPSSAHDSPFFTTNSGAPVWNNNSSLTVGTRGPILLEDYHLLEKLANFDRERIPERVVHARGASAKGFFEVTHDITQLTSADFLRGPGVQTPVI
CAT2        MDPYKYRPASSYNSPFFTTNSGAPVWNNNSSMTVGPRGPILLEDYHLVEKLANFDRERIPERVVHARGASAKGFFEVTHDISNLTCADFLRAPGVQTPVI
CAT3        MDPYKYRPSSAYNAPFYTTNGGAPVSNNISSLTIGERGPVLLEDYHLIEKVANFTRERIPERVVHARGISAKGFFEVTHDISNLTCADFLRAPGVQTPVI
            ****: **:*::::**:***.**** ** **:*:* ***:*******:**:*** ************* ************::**.*****.********
                                                                                                       200
CAT1        VRFSTVIHERGSPETLRDPRGFAVKFYTREGNFDLVGNNFPVFFVRDGMKFPDMVHALKPNPKSHIQENWRILDFFSHHPESLHMFSFLFDDLGIPQDYR
CAT2        VRFSTVIHERGSPETLRDPRGFAVKFYTREGNFDLVGNNFPVFFIRDGMKFPDMVHALKPNPKSHIQENWRILDFFSHHPESLNMFTFLFDDIGIPQDYR
CAT3        VRFSTVVHERASPETMRDIRGFAVKFYTREGNFDLVGNNTPVFFIRDGIQFPDVVHALKPNPKTNIQEYWRILDYMSHLPESLLTWCWMFDDVGIPQDYR
            ******:***.****:** ******************* ****:***::***:*********::*** *****::** ****  : ::***:*******
                                                                                                       300
CAT1        HMEGAGVNTYMLINKAGKAHYVKFHWKPTCGIKCLSDEEAIRVGGANHSHATKDLYDSIAAGNYPQWNLFVQVMDPAHEDKFDFDPLDVTKIWPEDILPL
CAT2        HMDGSGVNTYMLINKAGKAHYVKFHWKPTCGVKSLLEEDAIRVGGTNHSHATQDLYDSIAAGNYPEWKLFIQIIDPADEDKFDFDPLDVTKTWPEDILPL
CAT3        HMEGFGVHTYTLIAKSGKVLFVKFHWKPTCGIKNLTDEEAKVVGGANHSHATKDLHDAIASGNYPEWKLFIQTMDPADEDKFDFDPLDVTKIWPEDILPL
            **:* **:** ** *:**. :**********:* * :*:*  ***:******:**:*:**:****:*:**:* :***.************* ********
                                                                                                       400
CAT1        QPVGRLVLNKNIDNFFNENEQIAFCPALVVPGIHYSDDKLLQTRIFSYADSQRHRLGPNYLQLPVNAPKCAHHNNHHDGFMNFMHRDEEVNYFPSRLDPV
CAT2        QPVGRMVLNKNIDNFFAENEQLAFCPAIIVPGIHYSDDKLLQTRVFSYADTQRHRLGPNYLQLPVNAPKCAHHNNHHEGFMNFMHRDEEVNYFPSRYDQV
CAT3        QPVGRLVLNRTIDNFFNETEQLAFNPGLVVPGIYYSDDKLLQCRIFAYGDTQRHRLGPNYLQLPVNAPKCAHHNNHHEGFMNFMHRDEEINYYPSKFDPV
            *****:***:.***** *.**:** *.::****:******** *:*:*.*:*************************:**********:**:**: * *

                                                                                               492
CAT1        RHAEKYPTTPIVCSGNREKCFIGKENNFKQPGERYRSWDSDRQERFVKRFVEALSEPRVTHEIRSIWISY-SQADKSLGQKLATRLNVRPNF
CAT2        RHAEKYPTPPAVCSGKRERCIIEKENNFKEPGERYRTFTPERQERFIQRWIDALSDPRITHEIRSIWISYWSQADKSLGQKLASRLNVRPSI
CAT3        RCAEKVPTPTNSYTGIRTKCVIKKENNFKQAGDRYRSWAPDRQDRFVKRWVEILSEPRLTHEIRGIWISYWSQADRSLGQKLASRLNVRPSI
            * *** **..   :* * :*.* ******:.*:***:: .:**:**::*::: **:**:*****.***** ****:*******:******.:
```

B

```
         |         |         |         |         |         |         |         |         |
MANRGGCVTVAAEEMDELRRRNIELSREVAEMKTEMIKLWQRTVVAEEAEEQLCSQLAELEVESLEQARDYHDRMLFLMDQISRLSSSSVVSSS
```

Fig. S5. Amino acid sequence of the aligned CAT1, CAT2 and CAT3 (**A**) and of LSU1 (**B**). The residues highlighted in yellow are presumably involved in the CAT2-LSU1 interaction (based on the structural model shown in Figure 7). The residues highlighted in green are those different in CAT2 and CAT3 what perhaps might explain the observed difference in the Y2H interaction of the N-terminally truncated AD-CAT2Δ54 and AD-CAT3Δ54 variants with LSU1. CAT1 is shown only for the comparison and was not analysed in this study.

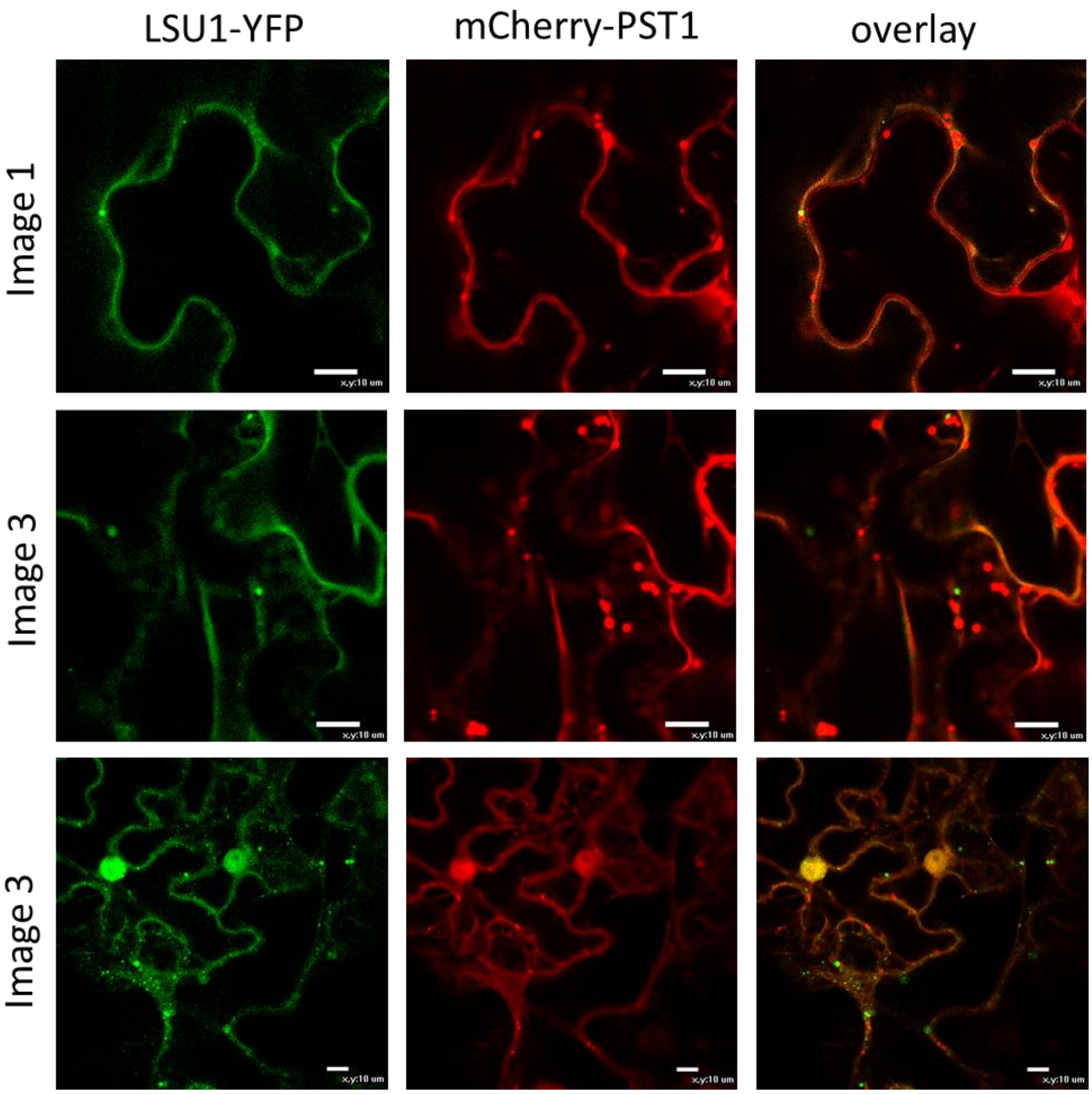

Fig. S6. Co-localization of LSU1-YFP with mCherry-PTS1 used as a peroxisomal marker. Three independent images represent three observed types of co-localization using in planta transient expression experiment. Upper slides – colocalization of both signals, middle slides – lack of any co-localization, bottom slides – co-localization in large structures, presumable large damaged peroxisomes.
